# Cation and voltage dependence of lidocaine inhibition of the hyperpolarization-activated cyclic nucleotide-gated HCN1 channel

**DOI:** 10.1038/s41598-017-01253-x

**Published:** 2017-04-28

**Authors:** Igor Putrenko, Raymond Yip, Stephan K. W. Schwarz, Eric A. Accili

**Affiliations:** 1 Department of Cellular and Physiological Sciences, The University of British Columbia, Vancouver, British Columbia Canada; 2 Department of Anesthesiology, Pharmacology & Therapeutics, The University of British Columbia, Vancouver, British Columbia Canada; 3 Department of Anesthesia, St. Paul’s Hospital/Providence Health Care, Vancouver, British Columbia Canada

## Abstract

Lidocaine is known to inhibit the hyperpolarization-activated mixed cation current (I_h_) in cardiac myocytes and neurons, as well in cells transfected with cloned Hyperpolarization-activated Cyclic Nucleotide-gated (HCN) channels. However, the molecular mechanism of I_h_ inhibition by this drug has been limitedly explored. Here, we show that inhibition of I_h_ by lidocaine, recorded from Chinese hamster ovary (CHO) cells expressing the HCN1 channel, reached a steady state within one minute and was reversible. Lidocaine inhibition of I_h_ was greater at less negative voltages and smaller current amplitudes whereas the voltage-dependence of I_h_ activation was unchanged. Lidocaine inhibition of I_h_ measured at −130 mV (a voltage at which I_h_ is fully activated) was reduced, and I_h_ amplitude was increased, when the concentration of extracellular potassium was raised to 60 mM from 5.4 mM. By contrast, neither I_h_ inhibition by the drug nor I_h_ amplitude at +30 mV (following a test voltage-pulse to −130 mV) were affected by this rise in extracellular potassium. Together, these data indicate that lidocaine inhibition of I_h_ involves a mechanism which is antagonized by hyperpolarizing voltages and current flow.

## Introduction

Lidocaine is a drug that depresses both sinus node activity and cardiac conduction, acts as a local anesthetic and topical analgesic, and, when applied systemically, produces central analgesia and sedation^1, 2^. In addition to its well-known action on voltage-gated sodium channels^3–5^, lidocaine may produce its therapeutic actions, as well its known adverse effects, by acting on other target proteins, including Hyperpolarization-activated Cyclic Nucleotide-gated (HCN) channels. For example, lidocaine has been shown to inhibit native HCN channels in cardiac Purkinje fibres^6, 7^, sinoatrial node pacemaker cells^8^, dorsal root ganglion neurons^9^, and thalamocortical neurons^10^, with an IC_50_ ranging from 38–99 µM and maximum inhibition varying from 70 to 100%. Two studies have examined the inhibition of heterologously expressed cloned HCN channels by lidocaine, with IC_50_ and maximum inhibition values of 67–276 µM and 42–44%, respectively^11, 12^. The molecular mechanism underlying the action of lidocaine on HCN channels and the basis for the variation in lidocaine’s effect among studies of native and cloned HCN channels remain unknown.

In this study, we examine the mechanistic basis of the inhibition of the HCN channel and associated hyperpolarization-activated current, I_h_, by lidocaine. Four HCN genes have been identified in mammals (HCN1-HCN4) and they are similar in primary sequence to the *Shaker*-related voltage-gated potassium (Kv) channels^13–15^. However, HCN channels have recently been shown to have a structure that is similar but not identical to those channels^16, 17^. Like Kv channels, HCN channels are tetrameric and each subunit is multi-membrane spanning and possesses an intracellular N and C-terminus^16^. However, the recently solved human HCN1 channel structure^16^ shows that the selectivity filter (the portion of the pore closest to the outside of the cell) of the HCN channel contains fewer binding sites for cations, which may help to explain the permeability to both sodium and potassium ions^13–15, 18^. The fourth S4 transmembrane segment, the voltage sensor, is long and appears to hold the pore (transmembrane S6 helices) in a compact and potentially closed conformation. Such a structural arrangement suggests that, unlike Kv channels^19^, the pore is naturally open at hyperpolarized potentials and that the voltage sensors apply pressure to close the pore during depolarization; this interpretation made from the HCN1 structure is consistent with that made previously by a combined functional and mutagenesis study of the HCN2 channel^20^, which is the isoform that is phylogenetically the closest to the HCN1 isoform^21^.

Here, we focus on lidocaine inhibition of the HCN1 isoform, which has been found throughout the brain and in the conduction system of the heart^22^ and has been shown in HCN1-knockout mice to contribute to the electrical and cellular activity of different types of cells, including sinoatrial node pacemaker myocytes^23^, somatosensory neurons^24^, cortical neurons^25^, cerebellar Purkinje neurons^26^, and hippocampal neurons^27^. We show that lidocaine inhibition of HCN1-mediated I_h_ is near complete within one minute of exposure, is reversible, and involves a mechanism which is antagonized by hyperpolarizing voltages and current flow.

## Materials and Methods

### Cell Culture and Transfection

Chinese hamster ovary (CHO) cells were maintained in Ham’s F-12 medium (Life Technologies, Burlington, ON, Canada) supplemented with 50 µg/ml penicillin/streptomycin (Life Technologies) and 10% fetal bovine serum (Life Technologies). Cells were grown on glass coverslips (22 × 22 mm) in 35 mm dishes at 37 °C with 5% CO_2_. Transfection of a mammalian expression vector encoding mouse HCN1 (pcDNA3, which was a kind gift from Martin Biel, Ludwig-Maximilians-Universität München, Germany) (2 µg per dish) was performed using the FuGene6 transfection reagent (Roche Diagnostics, Laval, QC, Canada) according to the manufacturer’s protocol. The DNA plasmid encoding Green Fluorescence Protein (GFP) was co-transfected for visualization of transfected cells.

### Electrophysiological Recordings

Approximately 24 to 48 hours post-transfection, a small piece of coverslip was paced into a recording chamber (900 µl) on the stage of an inverted microscope. Cells were perfused (0.5–1 ml/min) with a low potassium extracellular solution containing (mM): KCl, 5.4; NaCl, 135; MgCl_2_, 0.5; CaCl_2_, 1.8; HEPES, 5, and titrated to pH 7.4 with NaOH. It should be noted that “low” refers only to the other solutions used in this study (see below) whereas a concentration of 5.4 mM potassium in the extracellular solution is usually considered to be a normal concentration *in vivo*. Cells expressing GFP were selected for patch clamp recording. After attaining the whole-cell configuration, cells were perfused by using a Fast Step SF77B perfusion system (Warner Instruments, Hamden, CT), with either the same, low potassium extracellular solution or solutions containing different proportions of KCl and NaCl. The 30 and 60 mM potassium extracellular solutions contained 30 mM KCl and 110 mM NaCl and 60 mM KCl and 80 mM NaCl, respectively, and were titrated to pH 7.4 with either NaOH or KOH. The calculated osmolarities for the internal and external solutions used were ~290 mOsm. Patch pipettes were pulled from borosilicate glass (World Precision Instruments, Inc., Sarasota, FL) using a P-100 electrode puller (Sutter Instruments, Novato, CA) and filled with an intracellular solution containing (mM): KCl, 130; NaCl, 10; MgCl_2_, 0.5; EGTA, 1; HEPES, 5, adjusted to pH 7.4 with KOH, and had a resistance of 4–5 MΩ. Whole cell patch-clamp recordings were performed with an Axopatch 200B amplifier (Molecular Devices, LLC, Sunnyvale, CA) and a Digidata 1320A data acquisition system (Molecular Devices, LLC) using pCLAMP software (version 10, Molecular Devices, LLC). The membrane currents were low-pass filtered at a frequency of 2 kHz and digitized at 5 kHz. Data were collected more than 5–10 min after whole cell access to allow the internal pipette solution to equilibrate with the cell. Experiments were carried out at room temperature (21–23 °C).

In previous studies, we characterized aspects of HCN1 channel expression in CHO cells^28, 29^. By Western blotting and patch clamp electrophysiology, we showed that untransfected CHO cells do not express HCN channel protein or HCN-mediated currents. Upon transfection, robust I_h_ currents were elicited from GFP-expressing cells and HCN1 channel protein is abundant in Western blots. As we found previously, robust hyperpolarization-activated currents were observed in the current study when recording from cells that expressed GFP.

Lidocaine HCl was purchased from Sigma–Aldrich Canada Ltd. (Mississauga, ON, Canada), diluted in corresponding extracellular solution and applied by using the Fast Step SF77B perfusion system. Although the transfection efficiency was quite good, we recorded from only one cell per dish to avoid carrying out voltage protocols on cells that had already been exposed to the drug. New dishes were also employed if measurements were unfinished because of issues such as cells becoming unresponsive to voltage or large and irreversible alterations in access resistance or leak current. Lidocaine was perfused for 1–2 minutes in order to obtain steady state inhibition.

### Data Analysis

Data were analyzed using ORIGIN 8 (OriginLab Corporation, Northampton, MA). To determine the IC_50_ and Hill coefficient, concentration-response curves were normalized and fitted using the Hill equation as follows:

1 $$I/{I}_{{\rm{\max }}}={[{\rm{C}}]}^{h}/({[{{\rm{IC}}}_{50}]}^{h}+{[{\rm{C}}]}^{h})$$

where *I* represents the current measured in the presence of a given drug concentration; *I*_max_ is the control current measured in the absence of the drug; [C] is the drug concentration; IC_50_ is the half-maximal inhibitory concentration; and *h* is the Hill coefficient.

The normalized conductance-voltage relationship for steady-state activation of HCN1 current was fitted by the Boltzmann equation:

2 $$G/{G}_{{\rm{\max }}}=1/(1+\exp [(V\mbox{--}{V}_{50})/k])$$

where *G* is the conductance estimated after the current activation at a hyperpolarizing step [*V*]; *G*_max_ is the maximum conductance obtained after the most hyperpolarizing step; *V*_50_ is the half-maximal activation potential; and *k* is the slope factor.

To model the effect of extracellular potassium on inward steady-state I_h_, the relationship between concentration and current obtained from individual cells was fitted to the Michaelis-Menten model:

3 $${I}={{I}}_{{\rm{\max }}}{[{{\rm{K}}}^{+}]}_{{\rm{o}}}/({[{{\rm{K}}}^{+}]}_{{\rm{o}}}+{K}_{{\rm{app}}})$$

where *I* represents the current measured at a given potassium concentration, *I*_max_ is the maximum current, [K^+^]_o_ is the extracellular potassium concentration, and *K*_app_ is the apparent dissociation constant.

Reversal potentials in different concentrations of extracellular potassium were determined from leak-subtracted steady state current obtained from a hyperpolarizing pulse to −90 mV and current obtained from a subsequent pulse to −60 mV. For each cell, values for the steady state at −90 mV and for the subsequent tail current at −60 mV was plotted versus voltage and joined by a straight line, which was extrapolated to cross the x-axis to yield an estimate of reversal potential.

Data are presented as mean ± SEM. We used one-way ANOVA to test for comparisons of more than two groups. Comparisons between two groups were conducted with the use of a paired or unpaired Student *t* test; Statistical tests were two-tailed and results were considered significant at α = 0.05.

## Results

### Inhibition of HCN1-mediated I_h_ conductance by lidocaine is greater at less negative potentials

**Figure 1:**
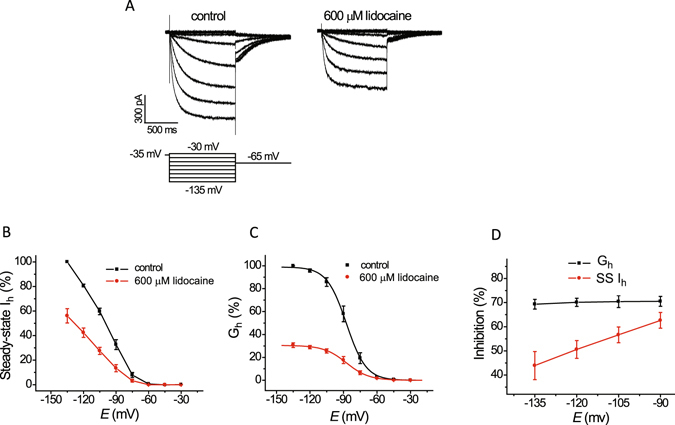
Lidocaine inhibits HCN1-mediated I_h_ conductance to a greater extent at less negative voltages but does not alter the voltage-dependence of I_h_ activation. (**A**) Representative traces of I_h_ activated by membrane voltages in the range from −30 mV to −135 mV at 30 mM extracellular potassium under control conditions and following application of 600 µM lidocaine. Activation voltage pulses were followed by a repolarization to −65 mV. (**B**) Plots of steady-state I_h_, before and after lidocaine, versus voltage, collected from current traces as shown in ‘**A**’; I_h_ amplitudes in the presence of lidocaine were normalized to those in the control solution lacking lidocaine. (**C**) Plots of G_h_, determined from tail current amplitudes at −65 mV as shown in ‘**A**’, before and after lidocaine, versus voltage; I_h_ amplitudes in the presence of lidocaine were normalized to those in the control solution lacking lidocaine. The solid curved line represents fitting of the values by a single exponential Boltzmann relation. Neither the mid-activation voltage (*V*_1/2_) of G_h_ nor the slope factor of this relationship was altered (n = 4; −87.2 ± 2.7 vs. −86.9 ± 3.6 mV and 8.5 ± 0.6 vs. 8.7 ± 0.6; paired *t*-test, *P* = 0.79 and *P* = 0.89, respectively). (**D**) Plots of the fraction of steady-state I_h_ and G_h_ inhibited by lidocaine versus voltage. Steady-state I_h_ inhibition increased from 43.9 ± 5.8% at −135 to 62.4 ± 3.3% at −90 mV (n = 4; ANOVA, *P* = 0.038) whereas G_h_ was not altered significantly (n = 4; 69.3 ± 2.0 versus 70.5 ± 2.1%; *P* = 0.98).

We examined the inhibition by lidocaine (600 µM) of steady-state I_h_ at different voltages and of G_h_, which was estimated as the magnitude of the tail I_h_ at a constant potential (−65 mV) in 30 mM extracellular potassium (Fig. 1A–D). This concentration of lidocaine was used because it is in the mid-range of concentrations for inhibition of I_h_ at 30 mM potassium (see below). The fraction of steady state I_h_ inhibited by lidocaine increased from ~44% at −135 mV to ~62% at −90 mV (Fig. 1B and D). Inhibition of G_h_ by lidocaine was constant at ~70% and, in contrast to steady state I_h_, changed little over the same range of voltages (Fig. 1C and D). The lack of change in G_h_ and increase in inhibition of steady state I_h_ at progressively less negative voltages in the same range suggest that lidocaine does not modify the voltage-dependence of I_h_ activation whereas its inhibition of I_h_ conductance increases at less negative potentials.

### Raising extracellular potassium increases I_h_ in HCN1-expressing CHO cells without altering the voltage-dependence of activation

We next examined the effect of increasing current flow on lidocaine inhibition of I_h_. In order to do this, we raised the proportion of extracellular potassium in order to increase current flow, which is one of two approaches used previously to examine how current flow impacts ivabradine inhibition of I_h_; inhibition of I_h_ by that drug is less at more negative potentials and, for I_h_ in the sinoatrial node (I_f_) and I_h_ associated with the HCN4 isoform, inhibition is reduced by an increase in current flow^30, 31^.

We first determined how raising extracellular potassium impacts I_h_ conductance and the voltage-dependence of I_h_ activation when measured in CHO cells expressing the HCN1 isoform. In addition to a depolarizing shift in reversal potential, raising extracellular potassium is known to increase maximum I_h_ conductance in both native tissue^18, 32–37^ as well as in cells that express cloned HCN channels^13, 15, 28, 38–40^. We previously examined this unusual potassium-induced increase in conductance of I_h_ in CHO cells expressing the HCN1 isoform by substituting potassium for sodium in the extracellular solution and thereby leaving unchanged the concentration of both the total and permeant cations^28^. We used the same approach here to first characterize the effect of extracellular cations on I_h_ conductance and voltage dependence of I_h_ activation, and then to examine the effects of extracellular cations on inhibition of I_h_ by lidocaine.

**Figure 2:**
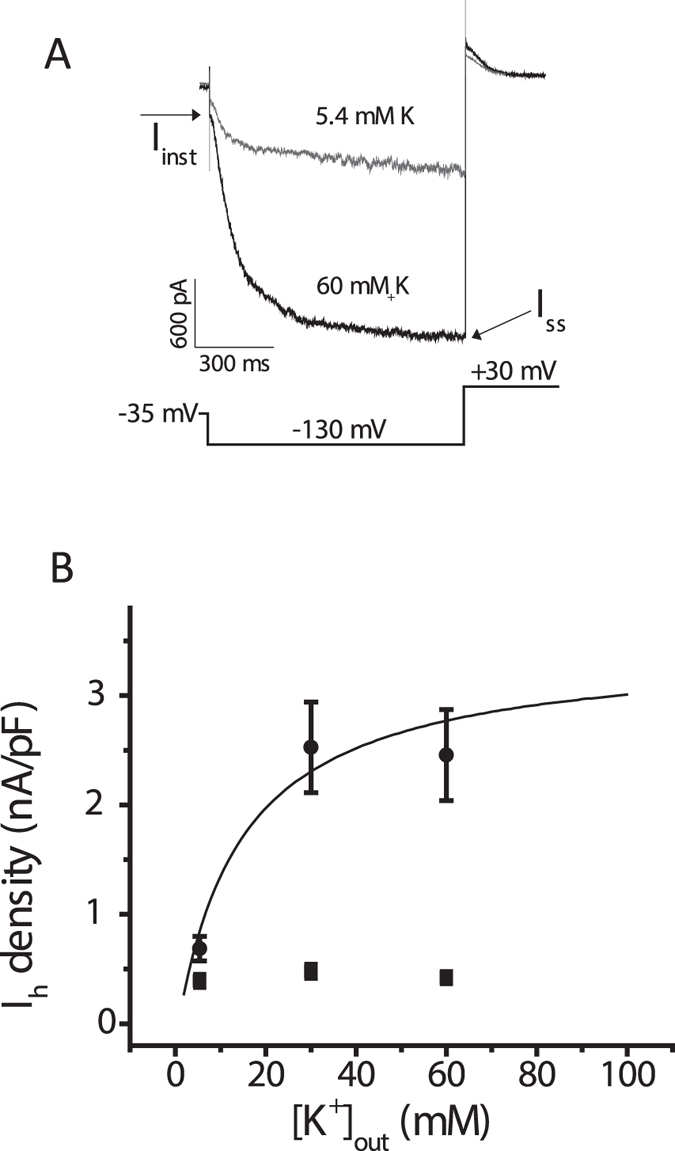
Raising extracellular potassium increases I_h_ amplitude at −130 mV in CHO cells that express the HCN1 isoform. (**A**) Representative traces of I_h_ elicited by the voltage protocol shown below them from the same cell exposed to extracellular potassium concentrations of 5.4 and 60 mM. The magnitude of I_h_ (for both inward current determined at −130 mV and outward current determined at +30 mV) was calculated as the difference between the instantaneous current at the beginning of each pulse (I_inst_) and the steady-state current (I_ss_) at the end of the pulse (*arrows*). Note the large difference in inward current measured at −130 mV and very little difference in current measured at +30 mV between the two extracellular solutions. (**B**) Amplitudes of I_h_ density (nA/pF) measured at −130 mV (inward current) and +30 mV (outward current), plotted as a function of the extracellular potassium concentration. Increasing extracellular K^+^ from 5.4 mM to 30 mM and to 60 mM produced a concentration-dependent increase in the average amplitude inward I_h_, which was measured at the end of the voltage pulse to −130 mV (filled circles; n = 22–32; ANOVA, *P* < 0.0001). The curved line represents a fit of the current density from individual cells to a Michaelis-Menten equation, yielding an apparent dissociation constant of 13.8 ± 7.1 mM. Maximum outward “tail” I_h_, which was measured 1 ms after the voltage step to +30 mV to minimize the contribution of capacitance current, was not significantly affected by raising the concentration of extracellular potassium (filled squares; *P* = 0.12). The reversal potentials for I_h_ in different concentrations of extracellular potassium and sodium were −26 ± 5 mV (5.4 mM Na, 135 mM K), −20 ± 4 mV (30 mM K, 110 mM Na), and −5 ± 9 mV (60 mM K, 80 mM Na); extracellular sodium concentrations were reduced proportionally to maintain osmolarity (see Materials and Methods).

Using patch clamp electrophysiology, we investigated inward I_h_ activated by a hyperpolarization to −130 mV and outward I_h_ recorded as the tail current at +30 mV (deactivation) from prior hyperpolarization to −130 mV. Increasing extracellular potassium from 5.4 mM to 60 mM produced a reversible increase in the magnitude of the inward I_h_, which reached a maximum in ~1 minute (Fig. 2). The dependence of the inward I_h_ on the extracellular potassium concentration was fitted to the Michaelis-Menten equation, yielding an apparent dissociation constant of ~14 mM. Our data are consistent with previous experiments on native and cloned HCNs showing that extracellular potassium has an activating effect on I_h_, which is separate from the changes in driving force. Outward I_h_ was minimally affected because raising extracellular potassium also depolarizes the reversal potential as shown in the original studies of the hyperpolarization-activated current^18, 33^. We have shown previously that raising potassium produces both a depolarizing shift in the reversal potential and an increase in slope conductance of the instantaneous I-V curve of I_h_ measured in CHO cells expressing the HCN1 isoform^28^.

**Figure 3:**
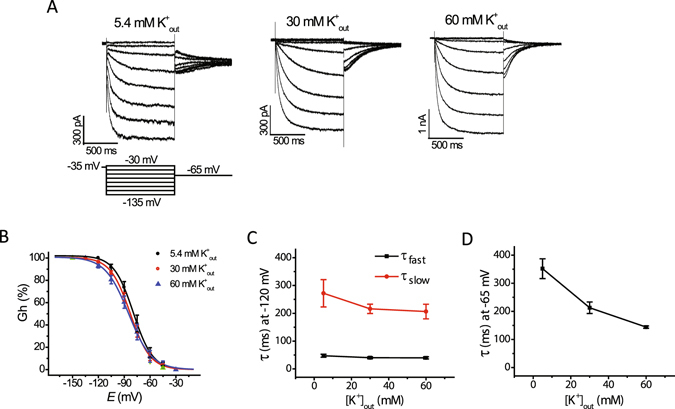
Raising extracellular potassium increases the rate of I_h_ deactivation without significantly affecting the voltage dependence and rate of I_h_ activation. (**A**) Representative traces of I_h_ activated by the voltage protocol shown below the traces at 5.4 mM, 30 mM, and 60 mM potassium in the extracellular recording solution. (**B**) Plots of normalized tail current amplitudes at −65 mV (G_h_) versus test voltage at the three concentrations of extracellular potassium. Curved lines represent fits to a Boltzmann function (*cf*. Material and Methods). Mid-activation voltages (V½) are −79.2 ± 3.6 mV, −82.2 ± 2.3 mV, and −84.7 ± 3.9 mV, at 5.4, 30, and 60 mM extracellular potassium, respectively, and were not significantly different (n = 7–11; ANOVA, *P* = 0.52). The corresponding values for slope factor (*k*) were 9.6 ± 0.5, 10.5 ± 0.7 and 12.4 ± 0.8, and were significantly different (n = 7–11; ANOVA, *P* = 0.045). (**C**) Plots of fast (*τ*_fast_) and slow (*τ*_slow_) time constants of activation of steady-state I_h_ measured at −120 mV as a function of extracellular potassium concentration. The values obtained at different potassium concentrations were not significantly different (n = 7–11; ANOVA, *P* = 0.42 (*τ*_fast_), *P* = 0.3 (*τ*_slow_)). (**D**) Plot of time constants of I_h_ deactivation as a function of the extracellular potassium concentration. The values of time constants of I_h_ deactivation was significantly different between values obtained at 5.4 mM, 30 mM and 60 mM extracellular potassium (n = 5–11; ANOVA, *P* < 0.001).

To examine the effect of extracellular potassium on the voltage dependence of I_h_ activation, we measured tail I_h_ amplitude at −65 mV (Fig. 3A) after a series of hyperpolarizing test voltages from a holding potential of −35 mV. Normalized tail current amplitude versus test voltage was plotted and fit with a Boltzmann equation to yield values for half-activation voltage (V_1/2_) and slope factor (*k*), as described in the Methods. Raising extracellular potassium did not significantly modify the range of I_h_ activation, although the slope factor was slightly increased (Fig. 3B). Thus, raising extracellular potassium increases the maximal conductance of I_h_ without altering the voltage-dependence of activation; its effect is the opposite of the effect of lidocaine on I_h_.

We next investigated the effect of extracellular potassium on the I_h_ activation rate, which was estimated by fitting the activation phase of the current with a double-exponential function. Neither the fast nor the slow time constant was affected by raising extracellular potassium (Fig. 3C). The time constant of I_h_ deactivation, which was determined by fitting the deactivating current with a single-exponential function (Fig. 3D), was significantly decreased by raising extracellular potassium.

### Elevation of extracellular potassium concentration opposes the effects of lidocaine on HCN1-mediated I_h_ amplitude measured at −130 mV

**Figure 4:**
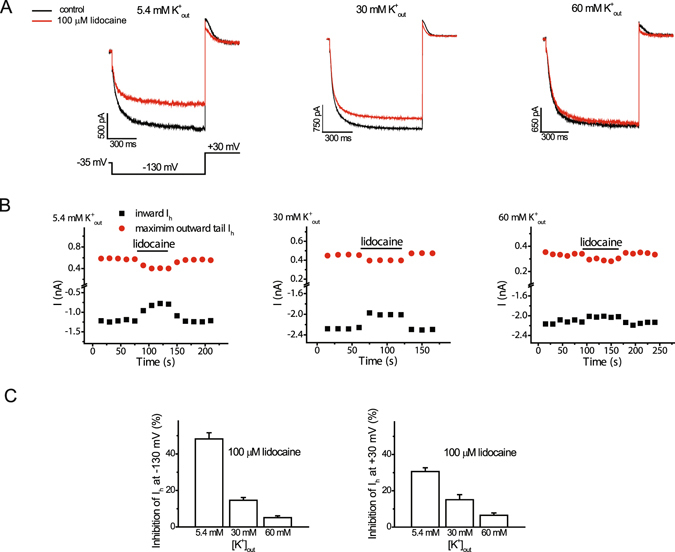
Elevation of extracellular potassium concentration opposes the effects of lidocaine on HCN1-mediated I_h_ amplitude. (**A**) Representative traces of inward steady-state and outward I_h_ under control conditions and following application of 100 µM lidocaine with a concentration of 5.4 mM, 30 mM, or 60 mM of potassium in the extracellular solution. Activation voltage pulses (1 s) producing inward steady-state current, followed by by a repolarization pulse to +30 mV to generate outward current, were applied with an interval of 15 s (**B**). Amplitudes of steady-state inward I_h_ (−130 mV) and maximum outward I_h_ (+30 mV) from individual cells with 5.4 mM, 30 mM, or 60 mM potassium in the extracellular solution under control conditions and in the presence of lidocaine (100 µM). The horizontal bar denotes the duration of lidocaine exposure. Note that the inhibition of inward and outward I_h_ by lidocaine was reversible. (**C**) Bar graphs of percent inhibition of I_h_ (left, −130 mV; right, +30 mV) by 100 mM lidocaine at different concentrations of extracellular potassium. Lidocaine produced reversible inhibition of I_h_ at −130 mV (inward) or +30 mV (maximum outward current) by 48.2 ± 3.4 and 30.2 ± 2.1%, respectively, (n = 6; paired *t*-test, *P* < 0.001) at 5.4 mM extracellular potassium. The percentage of I_h_ inhibited by this concentration of lidocaine was less at 30 and 60 mM extracellular potassium (n = 4–6; ANOVA, *P* < 0.001 at both voltages; n = 4–6 cells for each concentration).

We subsequently examined the influence of extracellular potassium on lidocaine inhibition of I_h_. In order to also assess the time it takes for inhibition by lidocaine, a hyperpolarizing voltage pulse to −130 mV (1 s) to activate I_h_, followed by a repolarizing pulse to +30 mV to deactivate the current (0.5 s), was applied every 15 seconds. Bath application of 100 µM lidocaine at 5.4 mM extracellular potassium reversibly inhibited inward I_h_ (at −130 mV) by ~48% (Fig. 4A,B). We noted close to complete inhibition of I_h_ by lidocaine after the first sweep of the protocol, within 15 s of application of the drug. The percentage of inward I_h_ (−130 mV) inhibited by 100 µM lidocaine was less at 30 and 60 mM extracellular potassium (Fig. 4C).

**Figure 5:**
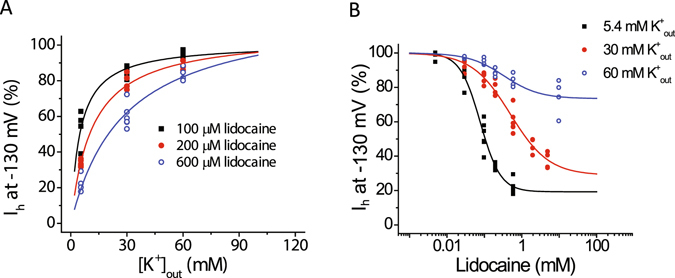
The concentration-response curve for the effects lidocaine and extracellular potassium on HCN1-mediated I_h_ at −130 mV demonstrates changes that are consistent with an interaction between them. (**A**) Plots of I_h_ amplitude at −130 mV versus concentration of extracellular potassium concentration in the presence of given concentrations of lidocaine (100, 200, and 600 µM). The solid lines represent fits to the Michaelis-Menten equation. An increase in the concentration of lidocaine from 100 to 600 µM resulted in an increase in the apparent dissociation constant of potassium from 5.3 ± 0.5 to 27.3 ± 8.9 mM (n = 4–6; ANOVA, *P* < 0.0001). (**B**) Plots of percent I_h_ inhibition at −130 mV versus lidocaine concentration. The solid line represents a fit using the Hill equation, which yielded values for IC_50_ of 78 ± 7, 495 ± 133, and 332 ± 151 µM (n = 4–6 cells; ANOVA, *P* < 0.0001) and estimated Hill coefficients of 1.6 ± 0.2, 0.84 ± 0.12, and 0.92 ± 0.3 at 5.4, 30, and 60 mM extracellular potassium, respectively. Lidocaine’s estimated maximum inhibition was also lower at the higher concentrations of extracellular potassium, declining from 80.7 ± 3.5 to 71.1 ± 5.9% and to 26.6 ± 3.4% upon raising the extracellular potassium concentration from 5.4 to 30 mM, and to 60 mM, respectively.

Next, we investigated how lidocaine impacts the concentration-response relationship for the effect of extracellular potassium on I_h_ at −130 mV. We plotted current amplitude remaining after exposure to a given concentration of lidocaine as a function of the extracellular potassium concentration (Fig. 5A). Fitting these plots with the Michaelis-Menten equation showed that an increase in the concentration of lidocaine from 100 to 600 µM resulted in an increase in the apparent dissociation constant of potassium from ~5.3 to ~27 mM.

We also sought to determine the effects of raising extracellular potassium on the concentration-response relationship for lidocaine inhibition of I_h_ at −130 mV. Fitting plots of percentage of inhibition versus lidocaine concentration with the Hill equation showed that raising the concentration of extracellular potassium reduced the potency of the drug at 30 mM potassium and both the potency and maximum inhibition of the drug at 60 mM potassium (Fig. 5B).

### Elevation of extracellular potassium concentration does not modify inhibition of HCN1-mediated I_h_ measured at +30 mV

Bath application of 100 µM lidocaine also reversibly inhibited outward I_h_ (measured as deactivating tail currents at +30 mV) (Fig. 4A). When I_h_ is compared near the beginning of the test voltage (determined at 1 ms, after the capacitive transient), the degree of inhibition is greater at lower levels of potassium (See Fig. 4C). However, inhibition of I_h_ at this voltage was complex and appeared to vary over the time as the current deactivated and the channel closed.

**Figure 6:**
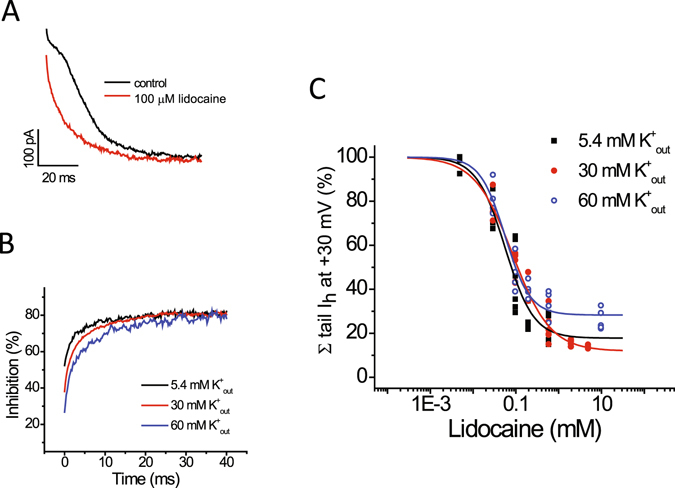
Lidocaine inhibition of HCN1-mediated I_h_ is unaltered at +30 mV by raising the concentration of extracellular potassium (**A**). Representative traces of outward tail I_h_ relaxation, recorded at +30 mV following hyperpolarization to −130 mV from a holding voltage of −35 mV, under control conditions and following application of 100 µM lidocaine, at 30 mM extracellular potassium. The tail currents are shown after 1 ms in order to remove any contribution from the capacitive transient and clarity. (**B**) Mean time courses of lidocaine (600 µM) inhibition of the outward I_h_ at different concentration of extracellular potassium (5.4, 30, and 60 mM) obtained by normalizing tail currents in the presence of lidocaine from those recorded in control solutions as shown in ‘**A**’. Time courses were fit with double exponential equations. The extent of inhibition at the beginning of the voltage pulse to +30 mV increased over the course of deactivation for each concentration of extracellular potassium. The initial value for inhibition was larger for the lower concentrations of extracellular potassium, and they reflected the mean values plotted in Fig. 4C. Maximum inhibition reached a plateau and was the same at all potassium concentrations (~70–80%). Raising extracellular potassium from 5.4 to 60 mM did not significantly alter either the fast or slow time constants of lidocaine block, which varied from 1.1 ± 0.13 to 1.33 ± 0.15 ms and from 8.1 ± 0.25 to 9.6 ± 0.9 ms (n = 4–5; ANOVA, *P* = 0.66 and 0.51), respectively, in the presence of 600 µM lidocaine. (**C**) Plots of normalized charge transfer versus lidocaine concentration at different extracellular potassium concentrations (5.4, 30, and 60 mM). To estimate the total charge transferred, the area under leak subtracted traces of tail I_h_ relaxation was calculated by integration and those calculated in the presence of lidocaine were normalized to those calculated in the absence of lidocaine. The solid line represents a fit using the Hill equation, which produced IC_50_ values of 56 ± 12, 84 ± 8, and 56 ± 7 µM (n = 4–6; ANOVA, *P* < 0.0001), Hill coefficients of 1.3 ± 0.3, 0.98 ± 0.11, and 1.49 ± 0.23, and maximum inhibitions of 82.2 ± 6.9, 88.1 ± 2.4, and 71.7 ± 2.4%, 5.4, 30, and 60 mM extracellular potassium, respectively. Values shown are those collected from individual experiments.

To determine how inhibition of current by lidocaine varied over time at +30 mV, the outward tail I_h_ in the presence of lidocaine was normalized to that measured under control conditions and fitting the obtained kinetics with a double-exponential function (Fig. 6A,B). The extent of inhibition differed over the course of the tail current; inhibition at the beginning of the voltage pulse to +30 mV increased over the course of deactivation for each concentration of extracellular potassium. The initial value for inhibition was larger for the lower concentrations of extracellular potassium, and they reflected the mean values plotted in Fig. 4C. However, the maximum inhibition reached a plateau and was the same at all potassium concentrations (~70–80%). Raising extracellular potassium from 5.4 to 60 mM did not significantly alter either the fast and slow time constants of inhibition in the presence of 600 µM lidocaine.

Lastly, we determined the degree of lidocaine inhibition of outward I_h_ at +30 mV by calculating the area under the current relaxation trace upon deactivation with the drug and normalizing this to the area under the relaxation curve in the absence of drug. We expected the degree of lidocaine inhibition of I_h_ determined in this way to reflect mainly the maximum inhibition reached after ~10 ms (~70–80%). Indeed, plotting the total normalized degree of inhibition of tail current area versus lidocaine concentration and fitting with the Hill plot produced three curves with similar values for maximum inhibition. Fitting also produced Hill coefficients of ~1 and values for IC_50_ of ~56, 87, and 56 μM. Values for maximum inhibition and IC_50_ are most similar to those values determined by lidocaine inhibition of I_h_ measured at −130 mV in 5.4 mM extracellular potassium.

## Discussion

In this study, we demonstrate that lidocaine inhibition of I_h_ in CHO cells that express the HCN1 channel depends upon the extracellular concentration of potassium and voltage. We show that inhibition of I_h_ is increased at less negative potentials whereas the dependence of I_h_ activation over the same range of voltages is not affected. We also show that the inhibition of I_h_ at −130 mV is reduced, and I_h_ conductance are increased, by raising potassium from 5.4 mM to 60 mM, whereas inhibition at +30 mV, and I_h_ conductance, are unaffected by the same changes in potassium. Thus, the data show that lidocaine inhibition of I_h_ and I_h_ conductance are inversely correlated and, together with concentration response curves, suggest that inhibition of I_h_ by lidocaine is antagonized by current flow and that the increase in current is antagonized by the drug. Finally, we show that lidocaine has no effect on the I_h_ activation curve.

Lidocaine, a weak base with a pK_a_ of approximately 7.9, exists in an uncharged basic form and a charged cationic form. As has been proposed for other channels^41^, the charged form may inhibit the HCN1 channel from the extracellular side or the uncharged form may diffuse through the plasma membrane, become re-protonated in the cytoplasm and inhibit the channel from the inside. Alternatively, the uncharged form of the drug may interact with the channel by traversing a lipophilic pathway in the plasma membrane towards a site on the channel which is embedded in the plasma membrane. Studies have shown that the application of QX-314, a quaternary derivative of lidocaine which is protonated and unable to cross the lipid bilayer, has no effect on I_h_ in neurons when applied on the external side whereas it inhibits I_h_ when applied from the intracellular side^9, 42, 43^. Thus, we currently favor the view that uncharged lidocaine diffuses through the plasma membrane, becomes re-protonated inside the cell and inhibits the HCN1 channel from the inside.

Our data on lidocaine are similar to previous data on the inhibition of I_h_ by other drugs. For example, inhibition of I_h_ by the drugs ZD7288, UL-FS49, DK-AH3, and ivabradine is reduced at more negative voltages and these molecules have only small or negligible effects on the voltage-dependence of I_h_ activation^44–48^. Like the lidocaine analog, QX-314, the aforementioned drugs are thought inhibit I_h_ in their cationic form by acting on a site which is accessible from the inside of the cell^31, 44, 45, 49^. Like lidocaine inhibition of I_h_ in our study, ivabradine inhibition of I_h_ in rabbit sino-atrial node cells and I_h_ in HEK cells heterologously expressing the HCN4 isoform is also increased when current is reduced; in those studies, current was reduced by reducing the proportion of extracellular potassium (as we did in our study) or by adding millimolar levels of extracellular cesium^30, 31^. In these studies, it was proposed that the dependence of ivabradine inhibition on current was due to its interaction with permeant ions on the inner aspect of the pore. Thus, together with the intracellular action of QX-314 on I_h_ in other systems, it seems possible that, in our study, the reduced inhibition of I_h_ by lidocaine at larger current amplitudes is due to its greater interaction with permeant ions on the inner side of the HCN1 pore. Further studies will be required to understand the interaction between current amplitude and lidocaine, and why drug potency is reduced mainly in 30 mM potassium whereas both potency and maximum inhibition of I_h_ by the drug are impacted by the drug in 60 mM potassium.

The present findings are important for understanding the myriad of effects of lidocaine *in vivo*. The greatest inhibition of I_h_ by lidocaine in our study occurred at the lowest concentration of extracellular potassium utilized, 5.4 mM, and the highest concentration of sodium (130 mM); these are close to the usual physiological concentrations for these cations. Our data in Fig. 1 imply that the inhibition of I_h_ by lidocaine increases progressively as the membrane potential becomes less negative suggesting that the effect of this drug might vary with membrane potential *in vivo* e.g. during an action potential. The concentration of potassium and sodium are also known to vary *in vivo*. In the heart, the concentration of extracellular potassium may range between 3 and 10 mM, and has been proposed to contribute to bradycardia that is mediated by HCN channels^50–52^. In the central nervous system, a range of potassium concentrations between 3 and 12 mM has been measured, while extracellular sodium may decrease by up to 7 mM during repetitive activity^53–55^. This variation in cation concentration encountered *in vivo* could influence the inhibition of I_h_ by lidocaine.

The results from our study and others support the probability that lidocaine exerts some its effects *in vivo* by acting on the HCN1 isoform. For example, knockout of the HCN1 isoform from somatosensory neurons in the mouse has been shown to reduce cold allodynia and to contribute to action potential generation (Momin *et al*. 2008). Thus, inhibition of HCN1 by lidocaine likely contributes to its analgesic effects, and it will be important to determine the extent to which lidocaine inhibition of HCN1 contributes to this drug’s *in vivo* actions and how inhibition is impacted by voltage and current. However, lidocaine inhibits three of the four mammalian HCN isoforms (HCN1 as well as HCN2 and HCN4), all of which are found in neurons and cardiomyocytes^11^. While it remains unknown if lidocaine inhibition of HCN2 and HCN4 is as sensitive to current and voltage as HCN1, there are noted differences in the inhibition of HCN1 and HCN4 isoforms by ivabradine, with only the latter being prominently current-dependent^31^. In brain slice whole-cell recordings of rat thalamocortical neurons, we previously found that lidocaine inhibition of I_h_ (100 μM) was not dependent on voltage and was comparatively potent (IC_50_ = 72 μM) and near complete; on the basis of the present findings, these differences could be explained by the relatively low extracellular potassium concentrations utilized (2.5 mM)^10^. Differences in lidocaine inhibition of I_h_ in thalamocortical neurons versus I_h_ in HCN1-expressing CHO cells could be due to the different HCN isoforms that are expressed in the former, thought to be mainly HCN2 and HCN4^56–60^, or to other factors such as other differences in recording conditions and cellular context.

In summary, the results of the present study show that lidocaine inhibition of I_h_ in CHO cells expressing the HCN1 isoform is reduced at hyperpolarizing voltages and inversely correlated with increases in I_h_ amplitude. Our data, together with data on lidocaine inhibition of I_h_ in other systems, suggest that lidocaine may diffuse through the plasma membrane, becomes re-protonated inside the cell and inhibits the channel by binding to the inner side of the HCN1 pore, where it interacts with permeant ions. Future studies will be required test this idea and to determine if inhibition occurs in the same way in other members of the HCN channel family and other members of the lidocaine family of drugs.

## References

[1] McCarthy GC, Megalla SA, Habib AS (2010). Impact of intravenous lidocaine infusion on postoperative analgesia and recovery from surgery: a systematic review of randomized controlled trials. Drugs.

[2] Vigneault L (2011). Perioperative intravenous lidocaine infusion for postoperative pain control: a meta-analysis of randomized controlled trials. Can J Anaesth.

[3] Hille B (1977). The pH-dependent rate of action of local anesthetics on the node of Ranvier. The Journal of general physiology.

[4] Strobel GE, Bianchi CP (1970). The effects of pH gradients on the action of procaine and lidocaine in intact and desheathed sciatic nerves. J Pharmacol Exp Ther.

[5] Bean BP, Cohen CJ, Tsien RW (1983). Lidocaine block of cardiac sodium channels. The Journal of general physiology.

[6] Weld FM, Bigger JT (1976). The effect of lidocaine on diastolic transmembrane currents determining pacemaker depolarization in cardiac Purkinje fibers. Circ Res.

[7] Carmeliet E, Saikawa T (1982). Shortening of the action potential and reduction of pacemaker activity by lidocaine, quinidine, and procainamide in sheep cardiac purkinje fibers. An effect on Na or K currents?. Circ Res.

[8] Rocchetti M, Armato A, Cavalieri B, Micheletti M, Zaza A (1999). Lidocaine inhibition of the hyperpolarization-activated current (I(f)) in sinoatrial myocytes. J Cardiovasc Pharmacol.

[9] Bischoff U, Brau ME, Vogel W, Hempelmann G, Olschewski A (2003). Local anaesthetics block hyperpolarization-activated inward current in rat small dorsal root ganglion neurones. British journal of pharmacology.

[10] Putrenko I, Schwarz SK (2011). Lidocaine blocks the hyperpolarization-activated mixed cation current, I(h), in rat thalamocortical neurons. Anesthesiology.

[11] Meng, Q. T., Xia, Z. Y., Liu, J., Bayliss, D. A. & Chen, X. Local Anesthetic Inhibits Hyperpolarization Activated Cationic Currents. *Mol Pharmacol* (2011). 10.1124/mol.110.070227 PMC3082936 21303986

[12] Tamura A (2009). Effects of antiarrhythmic drugs on the hyperpolarization-activated cyclic nucleotide-gated channel current. J Pharmacol Sci.

[13] Gauss R, Seifert R, Kaupp UB (1998). Molecular identification of a hyperpolarization-activated channel in sea urchin sperm. Nature.

[14] Santoro B (1998). Identification of a gene encoding a hyperpolarization-activated pacemaker channel of brain. Cell.

[15] Ludwig A, Zong X, Jeglitsch M, Hofmann F, Biel M (1998). A family of hyperpolarization-activated mammalian cation channels. Nature.

[16] Lee CH, MacKinnon R (2017). Structures of the Human HCN1 Hyperpolarization-Activated Channel. Cell.

[17] Long SB, Campbell EB, Mackinnon R (2005). Crystal structure of a mammalian voltage-dependent Shaker family K+ channel. Science.

[18] DiFrancesco D (1981). A study of the ionic nature of the pace-maker current in calf Purkinje fibres. The Journal of physiology.

[19] Yifrach O, MacKinnon R (2002). Energetics of pore opening in a voltage-gated K(+) channel. Cell.

[20] Macri V, Nazzari H, McDonald E, Accili EA (2009). Alanine scanning of the S6 segment reveals a unique and cAMP-sensitive association between the pore and voltage-dependent opening in HCN channels. J Biol Chem.

[21] Jackson HA, Marshall CR, Accili EA (2007). Evolution and structural diversification of hyperpolarization-activated cyclic nucleotide-gated channel genes. Physiol Genomics.

[22] Biel M, Wahl-Schott C, Michalakis S, Zong X (2009). Hyperpolarization-activated cation channels: from genes to function. Physiol Rev.

[23] Fenske S (2013). Sick sinus syndrome in HCN1-deficient mice. Circulation.

[24] Momin A, Cadiou H, Mason A, McNaughton PA (2008). Role of the hyperpolarization-activated current Ih in somatosensory neurons. The Journal of physiology.

[25] Huang Z (2011). Presynaptic HCN1 channels regulate Cav3.2 activity and neurotransmission at select cortical synapses. Nature neuroscience.

[26] Nolan MF (2003). The hyperpolarization-activated HCN1 channel is important for motor learning and neuronal integration by cerebellar Purkinje cells. Cell.

[27] Kim CS, Chang PY, Johnston D (2012). Enhancement of dorsal hippocampal activity by knockdown of HCN1 channels leads to anxiolytic- and antidepressant-like behaviors. Neuron.

[28] Macri V, Accili EA (2004). Structural elements of instantaneous and slow gating in hyperpolarization-activated cyclic nucleotide-gated channels. J Biol Chem.

[29] Hegle AP, Nazzari H, Roth A, Angoli D, Accili EA (2010). Evolutionary emergence of N-glycosylation as a variable promoter of HCN channel surface expression. Am J Physiol Cell Physiol.

[30] Bucchi A, Baruscotti M, DiFrancesco D (2002). Current-dependent block of rabbit sino-atrial node I(f) channels by ivabradine. The Journal of general physiology.

[31] Bucchi A, Tognati A, Milanesi R, Baruscotti M, DiFrancesco D (2006). Properties of ivabradine-induced block of HCN1 and HCN4 pacemaker channels. The Journal of physiology.

[32] Mayer ML, Westbrook GL (1983). A voltage-clamp analysis of inward (anomalous) rectification in mouse spinal sensory ganglion neurones. The Journal of physiology.

[33] DiFrancesco D (1982). Block and activation of the pace-maker channel in calf purkinje fibres: effects of potassium, caesium and rubidium. The Journal of physiology.

[34] Edman A, Grampp W (1989). Ion permeation through hyperpolarization-activated membrane channels (Q-channels) in the lobster stretch receptor neurone. Pflugers Arch.

[35] Hestrin S (1987). The properties and function of inward rectification in rod photoreceptors of the tiger salamander. The Journal of physiology.

[36] Wollmuth LP, Hille B (1992). Ionic selectivity of Ih channels of rod photoreceptors in tiger salamanders. The Journal of general physiology.

[37] Solomon JS, Nerbonne JM (1993). Hyperpolarization-activated currents in isolated superior colliculus-projecting neurons from rat visual cortex. The Journal of physiology.

[38] Moroni A (2000). Kinetic and ionic properties of the human HCN2 pacemaker channel. Pflugers Arch.

[39] Ishii TM, Takano M, Xie LH, Noma A, Ohmori H (1999). Molecular characterization of the hyperpolarization-activated cation channel in rabbit heart sinoatrial node. J Biol Chem.

[40] Macri V, Proenza C, Agranovich E, Angoli D, Accili EA (2002). Separable gating mechanisms in a Mammalian pacemaker channel. J Biol Chem.

[41] Hille B (1977). Local anesthetics: hydrophilic and hydrophobic pathways for the drug-receptor reaction. The Journal of general physiology.

[42] Perkins KL, Wong RK (1995). Intracellular QX-314 blocks the hyperpolarization-activated inward current Iq in hippocampal CA1 pyramidal cells. Journal of neurophysiology.

[43] Kilb W, Luhmann HJ (2000). Characterization of a hyperpolarization-activated inward current in Cajal-Retzius cells in rat neonatal neocortex. Journal of neurophysiology.

[44] Shin KS, Rothberg BS, Yellen G (2001). Blocker state dependence and trapping in hyperpolarization-activated cation channels: evidence for an intracellular activation gate. The Journal of general physiology.

[45] Bois P, Bescond J, Renaudon B, Lenfant J (1996). Mode of action of bradycardic agent, S 16257, on ionic currents of rabbit sinoatrial node cells. British journal of pharmacology.

[46] Van Bogaert PP, Pittoors F (2003). Use-dependent blockade of cardiac pacemaker current (If) by cilobradine and zatebradine. European journal of pharmacology.

[47] Van Bogaert PP, Goethals M, Simoens C (1990). Use- and frequency-dependent blockade by UL-FS 49 of the if pacemaker current in sheep cardiac Purkinje fibres. European journal of pharmacology.

[48] BoSmith RE, Briggs I, Sturgess NC (1993). Inhibitory actions of ZENECA ZD7288 on whole-cell hyperpolarization activated inward current (If) in guinea-pig dissociated sinoatrial node cells. British journal of pharmacology.

[49] Van Bogaert PP, Goethals M (1992). Blockade of the pacemaker current by intracellular application of UL-FS 49 and UL-AH 99 in sheep cardiac Purkinje fibers. European journal of pharmacology.

[50] Paterson DJ (1996). Antiarrhythmic mechanisms during exercise. J Appl Physiol (1985).

[51] Choate JK, Nandhabalan M, Paterson DJ (2001). Raised extracellular potassium attenuates the sympathetic modulation of sino-atrial node pacemaking in the isolated guinea-pig atria. Exp Physiol.

[52] Kleber AG (1983). Resting membrane potential, extracellular potassium activity, and intracellular sodium activity during acute global ischemia in isolated perfused guinea pig hearts. Circ Res.

[53] Dietzel I, Heinemann U, Hofmeier G, Lux HD (1982). Stimulus-induced changes in extracellular Na+ and Cl− concentration in relation to changes in the size of the extracellular space. Exp Brain Res.

[54] Sykova E, Kriz N, Preis P (1983). Elevated extracellular potassium concentration in unstimulated spinal dorsal horns of frogs. Neurosci Lett.

[55] Sykova E (1983). Extracellular K+ accumulation in the central nervous system. Prog Biophys Mol Biol.

[56] Whitaker GM, Angoli D, Nazzari H, Shigemoto R, Accili EA (2007). HCN2 and HCN4 isoforms self-assemble and co-assemble with equal preference to form functional pacemaker channels. J Biol Chem.

[57] Abbas SY, Ying SW, Goldstein PA (2006). Compartmental distribution of hyperpolarization-activated cyclic-nucleotide-gated channel 2 and hyperpolarization-activated cyclic-nucleotide-gated channel 4 in thalamic reticular and thalamocortical relay neurons. Neuroscience.

[58] Ying SW (2007). Dendritic HCN2 channels constrain glutamate-driven excitability in reticular thalamic neurons. J Neurosci.

[59] Seifert R (1999). Molecular characterization of a slowly gating human hyperpolarization-activated channel predominantly expressed in thalamus, heart, and testis. Proc Natl Acad Sci USA.

[60] Ludwig A (2003). Absence epilepsy and sinus dysrhythmia in mice lacking the pacemaker channel HCN2. Embo J.

